# Study of stiffness and bearing capacity degradation of reinforced concrete beams under constant-amplitude fatigue

**DOI:** 10.1371/journal.pone.0192797

**Published:** 2018-03-09

**Authors:** Fangping Liu, Jianting Zhou, Lei Yan

**Affiliations:** 1 School of Civil Engineering, Chongqing Three Gorges University, Wanzhou, Chongqing, People’s Republic of China; 2 Engineering Research Center of Bridge Structure and Material in the Mountainous Area, Chongqing Jiaotong University, Nan’an, Chongqing, People’s Republic of China; Universidade de Lisboa, PORTUGAL

## Abstract

For a reinforced concrete beam subjected to fatigue loads, the structural stiffness and bearing capacity will gradually undergo irreversible degeneration, leading to damage. Moreover, there is an inherent relationship between the stiffness and bearing capacity degradation and fatigue damage. In this study, a series of fatigue tests are performed to examine the degradation law of the stiffness and bearing capacity. The results pertaining to the stiffness show that the stiffness degradation of a reinforced concrete beam exhibits a very clear monotonic decreasing "S" curve, i.e., the stiffness of the beam decreases significantly at the start of the fatigue loading, it undergoes a linear decline phase in the middle for a long loading period, and before the failure, the bearing capacity decreases drastically again. The relationship between the residual stiffness and residual bearing capacity is determined based on the assumption that the residual stiffness and residual bearing capacity depend on the same damage state, and then, the bearing capacity degradation model of the reinforced concrete beam is established based on the fatigue stiffness. Through the established model and under the premise of the known residual stiffness degradation law, the degradation law of the bearing capacity is determined by using at least one residual bearing capacity test data, for which the parameters of the stiffness degradation function are considered as material constants. The results of the bearing capacity show that the bearing capacity degradation of the reinforced concrete beam also exhibits a very clear monotonic decreasing "S" curve, which is consistent with the stiffness degradation process and in good agreement with the experiment. In this study, the stiffness and bearing capacity degradation expressions are used to quantitatively describe their occurrence in reinforced concrete beams. In particular, the expression of the bearing capacity degradation can mitigate numerous destructive tests and save cost. The stiffness and bearing capacity degradation expressions for a reinforced concrete beam can be used to predict the deformation and bearing capacity of a structure during the service process and determine the structural fatigue damage and degree of degradation.

## 1. Introduction

A reinforced concrete beam has the advantages of a low cost and simple construction process, and it is the main type beam that was used in the early construction of existing bridges. However, with the repeated loading of the fatigue load, the stiffness and bearing capacity will undergo a dynamic degradation. The degradation is gradual, and to some extent, the beam will be damaged. However, this type of destruction is not predictable and random, and its consequences are very serious [1–3]. This is a prominent problem in the field of disaster prevention and mitigation in engineering structures, and therefore, is of tremendous interest to researchers [4–6].

The stiffness test of a reinforced concrete beam is simple and easy, and there are numerous fatigue experiments that have been proposed over time. However, the results are only limited to the description of the stiffness degradation, though the study of the degradation law is not sufficient [7–9]. An experimental study of the bearing capacity degradation of reinforced concrete beams under fatigue loading is required to produce significant amount of data, but it demands tremendous manpower and material and financial resources and is subject to the experimental conditions, so that only very limited data are obtained. Therefore, such experimental studies are few. Both the stiffness degradation and bearing capacity degradation of reinforced concrete beams reflect the degree of damage to some extent. We can obtain the law of stiffness degradation under a fatigue load, and consider it as an index to determine the degree of damage of the beam. Following this, by identifying the relationship between the stiffness damage and bearing capacity damage, the research on the bearing capacity degradation of reinforced concrete beams can become simple and easy and save manpower, material resources, and financial costs, which is of tremendous practical significance.

Based on the above analysis, in this study, constant-amplitude fatigue tests are conducted for five reinforced concrete rectangular beams, the stiffness degradation parameters under the action of fatigue loading are examined, and the formula for calculating the stiffness degradation is obtained. Accordingly, the calculation formula for the bearing capacity degradation of the test beam is established. Finally, a quantitative description of the stiffness and bearing capacity degradation is realized, and consequently, numerous destructive tests are avoided. Using the stiffness and bearing capacity degradation formulas for reinforced concrete beams, the deformation and bearing capacity change of a structure in the service period can be predicted. They can also be used to determine the structural fatigue damage and degree of performance degradation.

## 2. Experimental design

### 2.1. Experimental overview

In this work, to study the degradation of the stiffness and bearing capacity of reinforced concrete beams under constant-amplitude fatigue loading, five reinforced concrete rectangular beams were fabricated in the laboratory. The experimental beams were divided into two groups. Static load tests were performed on the first group (one beam), and amplitude fatigue tests were conducted on the second group (four beams). All the experiments were performed on a 250-kN tonnage fatigue test machine in the structural laboratory of the Chongqing Jiaotong University.

### 2.2. Experimental materials and specimen design

#### 2.2.1 Concrete

The cement used in the experiment is P.O32.5. The sand is Chongqing local fine sand. The lithotripsy is limestone with a particle size of 5 mm to 10 mm. The test water is tap water. There is no admixture in the concrete. The test ratio is cement (No. 42.5 ordinary Portland cement):sand:gravel:water = 1:1.11:2.06:0.4, and the amount of cement in each cubic concrete is 525 kg. The concrete strength is 30 MPa. Six blocks with a size of 150 mm × 150 mm × 150 mm are poured. Three of these blocks are used to test the splitting tensile strength, and the other three are used to test the cube compressive strength. Additional six blocks with a size of 150 mm × 150 mm × 300 mm are poured. Three of these blocks are used to test the axial compressive strength, and the other three are used to test the elastic modulus. The average experimental values are listed in Table 1.

**Table 1:** Concrete parameter values.

Cube Compressive Strength (MPa)	Axial Compressive Strength (MPa)	Splitting Tensile Strength (MPa)	Elastic Modulus (MPa)
35.2	29.6	1.87	2.8×10^4^

#### 2.2.2 Steel

The mechanical properties of the materials directly affect the fatigue performance of a test beam. To eliminate this effect, the longitudinal reinforcement, stirrups, and ribs used in this experiment are all hot-rolled steel bars produced by the Chongqing Iron and Steel (Group) Limited Liability Company, and the steel used in this test are all products of the same batch. The longitudinal steel used in the beams is HRB335-grade threaded bar with a diameter of 20 mm. The stirrups and erect ribs are HPB300 round bars with a diameter of 8 mm. The mechanical properties of the longitudinal bars, as listed in Table 2, are tested by a WE-1000 hydraulic universal testing machine.

**Table 2:** Longitudinal steel parameter values.

Diameter Specification (mm)	Yield Strength(MPa)	Ultimate Strength (MPa)	Elongation (%)	Elastic Modulus (MPa)
20	439	554	18.9	2.13×10^5^

#### 2.2.3 Specimen design

**Fig 1:**
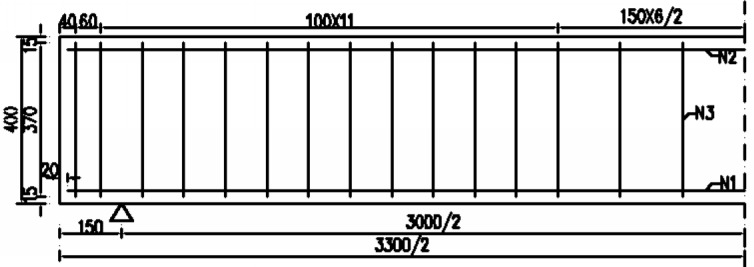
Size and reinforcement of the experimental beam.

The reinforced concrete beam used in the experiment is a rectangular solid section, with a size of 200 mm (width) × 400 mm (height) and beam length of 3300 mm (net span 3000 mm). The net protective layer thickness of the concrete is 20 mm. The size and reinforcement of the reinforced concrete beams are shown in Fig 1.

### 2.3 Test conditions

**Fig 2:**
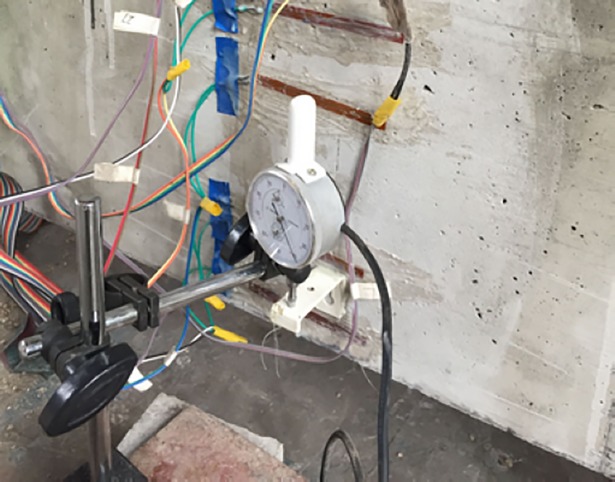
Schematic of the deflection test.

The experiment mainly tests the development of and change in the deflection of the beams with numerous fatigue cycles. The deflection at the mid-span and loading point is observed by setting dial gauges simultaneously, and to balance the error caused by the support displacement, a dial gauge is also arranged at each side of the beam at the two concrete supports. The method of setting and testing is as follows: on one side of the lower edge of the beam, L shaped plastic small parts are pasted for placing the dial gauges. To prevent damage to the dial gauges, they are removed when a measurement is not needed. The dial gauges are placed during the test, and the pointer is sufficiently compressed, causing it to move. The schematic of the deflection test of the experimental beam is shown in Fig 2.

The experimental beam numbers are labelled as JL-1 to JL-5. JL-1 beam is used in the static test to determine the ultimate load required in the fatigue experiment, and JL-2 to JL-4 beams are used in the fatigue tests under different load levels to determine the fatigue deflection information throughout the life cycle. To obtain the residual bearing capacity of JL-5 beam based on the fatigue life of JL-4 beam, a static loading test of JL-5 beam is performed when the fatigue cycle is approximately 0.8 times the life. The specific experimental conditions are listed in Table 3.

**Table 3:** Test conditions.

Beam No.	Loading System			Loading Instructions
JL-1	Static Load Test			Obtain cracking load p_cr_ and ultimate load P_u_
	Fatigue Lower Limit (P_min_)	Fatigue Upper Limit (P_max_)	Amplitude (ΔP)	Lower limit, upper limit, and amplitude of fatigue test
JL-2	0.1P_u_	0.6P_u_	0.5P_u_	Load to destruction with fatigue load
JL-3	0.1P_u_	0.7P_u_	0.6P_u_	
JL-4	0.1P_u_	0.8P_u_	0.7P_u_	
JL-5	0.1P_u_	0.8P_u_	0.7P_u_	Load to destruction with static load when cycle to 0.8 times life

### 2.4 Experimental device and loading method

#### 2.4.1 Experimental device

**Fig 3:**
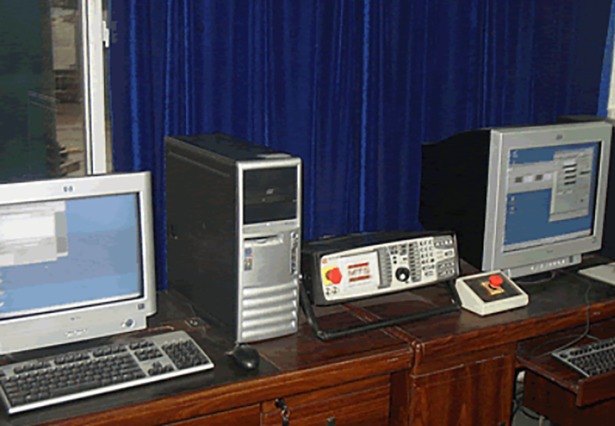
Testing machine control system.

Both the static load test and fatigue test are conducted using the online mode of a computer and the electro-hydraulic servo loading equipment. The maximum working range of the loading actuator used in this experiment is 250 kN, and the maximum working stroke is 75 mm. The test machine control system is shown in Fig 3.

#### 2.4.2 Loading method

**2.4.2.1 Static load test.**The objectives of the static load test are to determine cracking load P_cr_ and ultimate load P_u_ and decide whether the reinforcements of the beams are suitable. Before the formal static load test, the beams are first pre-loaded thrice. The actual static load test starts after the completion of the pre-load test. The static load test is conducted by grading the load with a load increment of 20 kN.

**Fig 4:**
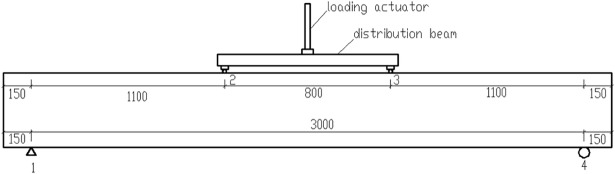
Loading schematic.

**Fig 5:**
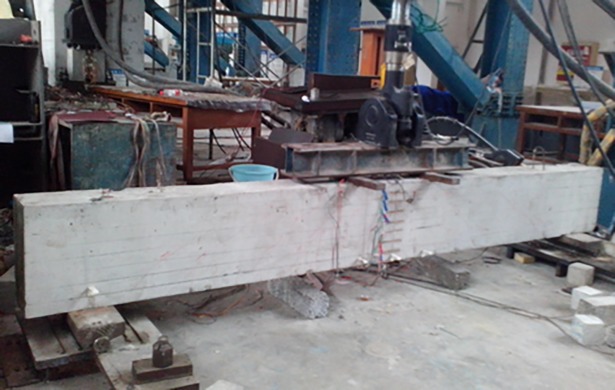
Fatigue test loading site.

**2.4.2.2 Fatigue load test.** To examine that the data acquisition system is working properly and the testing load control system is safe and reliable, the beams are also first pre-loaded thrice before the actual fatigue test. Because the test is controlled by the load amplitude, first, load (*P*_max_ + *P*_min_)/2 in the form of a graded load is tested, then the fatigue tests start after setting the amplitude to (*P*_max_ − *P*_min_)/2 and entering the load frequency and number of the next cycle. To prevent the occurrence of accidents, before the next phase of the experiment, the limits of the protection of the displacement and force are set to stop the loading system. The fatigue test is first stopped when the fatigue cycle ranges to 1 time, 1000 times, 10000 times, 20000 times, 50000 times, 100000 times, 200000 times, and every 200000 times thereafter. Then the dial gauges are placed appropriately, and it is ensured that the pointer and L type small part pasted on the beam side are in complete contact. Subsequently, the load is unloaded to zero, and the acquisition device is adjusted to zero. Next, the displacement information of the structure is collected under the zero state, and the load is loaded at intervals of 20 kN to the upper limit of fatigue load *P*_max_ and the displacement at each instant is measured. Finally, the dial gauges are removed, and the load is gradually loaded to (*P*_max_ − *P*_min_)/2 to continue the experiment. In this process, the initial loading from zero to *P*_max_ is considered as the first fatigue cycle. The loading frequency is 5 Hz. The loading schematic is shown in Fig 4, and the fatigue test loading site is presented in Fig 5.

## 3. Experiment result

**Fig 6:**
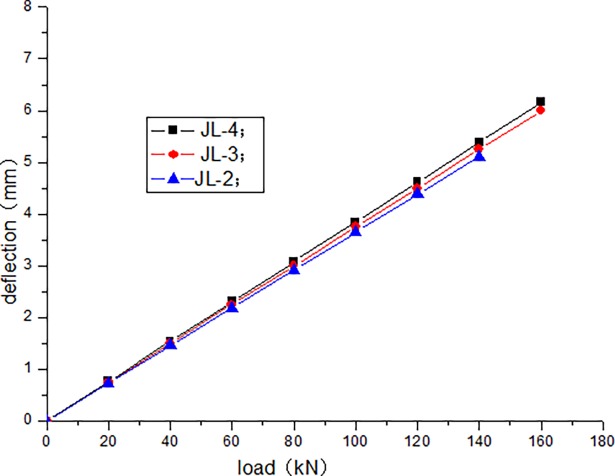
Load–deflection curve after 1 cycle.

**Fig 7:**
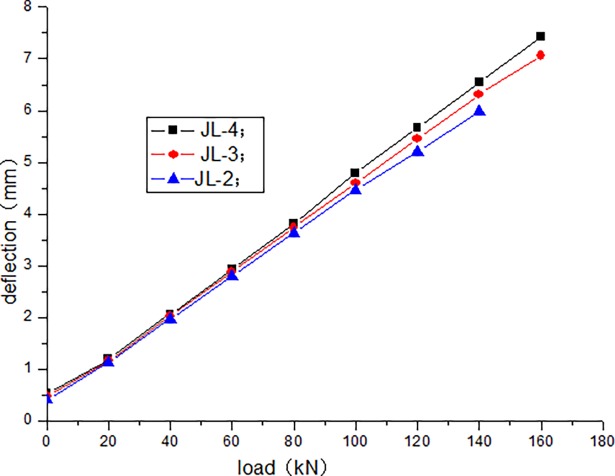
Load–deflection curve after 1000 cycles.

**Fig 8:**
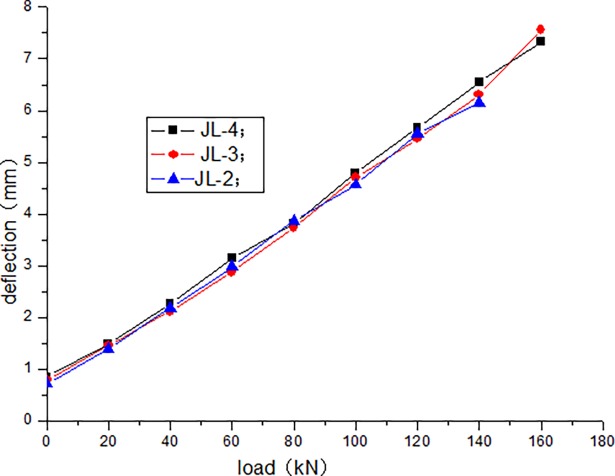
Load–deflection curve after 10000 cycles.

**Fig 9:**
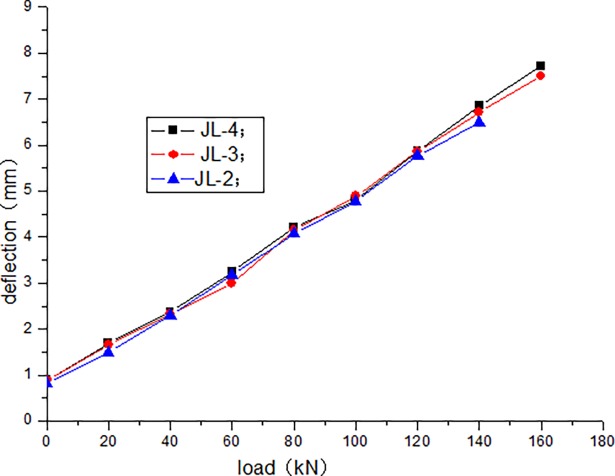
Load–deflection curve after 20000 cycles.

**Fig 10:**
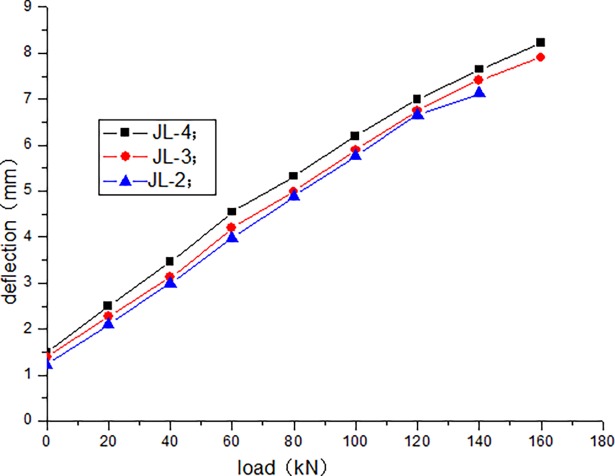
Load–deflection curve after 50000 cycles.

**Fig 11:**
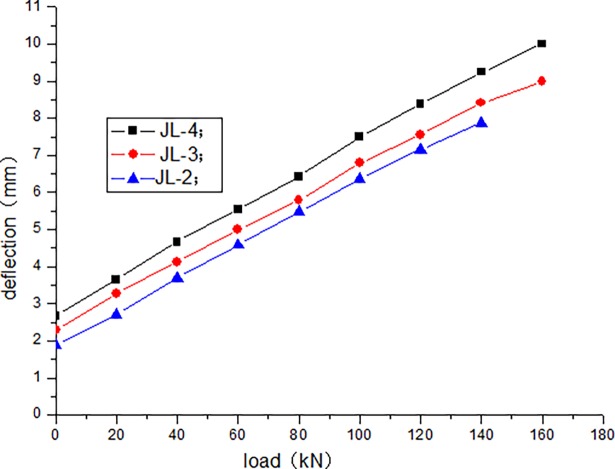
Load–deflection curve after 100000 cycles.

**Fig 12:**
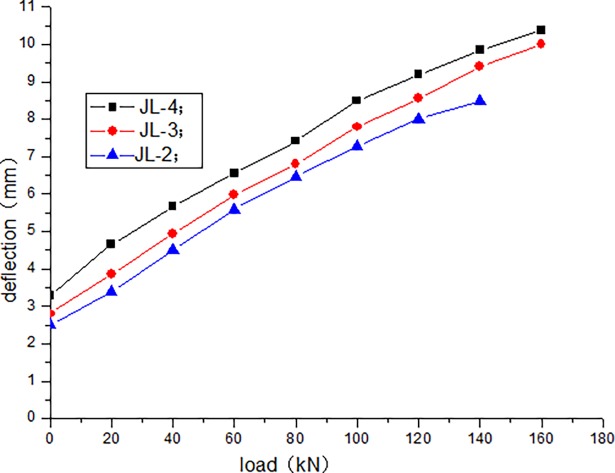
Load–deflection curve after 200000 cycles.

The cracking load of JL-1 beam obtained by static load test is 80 kN, and the ultimate load is 220 kN. The fatigue life of JL-2–JL-4 beams are as follows: 1680000 times, 1260000 times, and 640000 times, respectively. The static loading test of JL-5 beam is conducted after 500000 times of the fatigue loading, and the bearing capacity is 180 kN. During the test, the deflection of the beam in the upper limit of the fatigue load increases with the increase in the number of fatigue cycles. Table 4 presents the deflection of the beam in the upper limit of the fatigue load after the different cycle times. Figs 6–12 depict the relationship between the deflection and load of each beam in different cycle times.

**Table 4:** Mid-span deflection in *P*_max_ load after different cyclic times (mm).

beam	JL-2 (22 kN–132 kN)									
circle	1	1000	10000	20000	50000	100000	200000	400000	600000	800000
deflection	5.12	5.82	6.24	6.68	7.32	7.88	8.46	9.02	9.65	10.12
circle	1000000	1200000	1400000	1600000	1680000					
deflection	10.56	11.03	11.88	13.24	13.74					
beam	JL-3 (22 kN–154 kN)									
circle	1	1000	10000	20000	50000	100000	200000	400000	600000	800000
deflection	6.18	6.86	7.45	8.13	9.02	9.84	10.33	10.98	11.54	12.00
circle	1000000	1200000	1260000							
deflection	12.78	13.20	13.36							
beam	JL-4 (22 kN–176 kN)									
circle	1	1000	10000	20000	50000	100000	200000	400000	600000	620000
deflection	7.10	7.88	8.45	9.04	10.45	10.92	11.58	12.24	13.82	14.88

It can be seen from Figs 6 and 7 that, when the initial cyclic loading and fatigue loading are 1000 times, the relationship between the mid-span deflection of the beam and load increase is linear, and the effect of the load amplitude and fatigue is not obvious. It can be seen from Figs 8–12 that the starting point of the curve moves upward, indicating that the residual deflection increases rapidly. The three curves slowly start to disperse, and under the same load, the deflection of the beam at a larger loading level is relatively large. The results show that the effect of the fatigue load level is more clear with the increase in the fatigue time.

## 4. Stiffness degradation law

### 4.1 Definition of fatigue stiffness

Because of the degradation of material properties under fatigue loading, the deformation of a reinforced concrete beam increases monotonously and stiffness decreases monotonously with the increase in the fatigue cycle times. Therefore, to study the stiffness of reinforced concrete beams under fatigue loading, it most important to determine its degradation laws as a function of the fatigue load cycle number.

If, under the fatigue load, the initial stiffness of the beam is *B*_*s*_ and failure stiffness is *B*_*Nr*_, then during fatigue loading, the stiffness of the beam will decrease by *B*_*s*_ − *B*_*Nr*_. If the stiffness of a reinforced concrete beam degrades according to a function related to the fatigue life ratio, then the associated stiffness calculation formula can be obtained when the fatigue load acts n times. The formula is as follows:

$$B_{nr}=B_{s}-\left(B_{s}-B_{Nr}\right)\eta \left(\frac{n}{N}\right)$$ (1)

Dividing both sides of (1) by *B*_*s*_ yields

$$\frac{B_{nr}}{B_{s}}=1-\left(1-\frac{B_{Nr}}{B_{s}}\right)\eta \left(\frac{n}{N}\right)$$ (2)

In formulas (1) and (2), *B*_*s*_ is the initial stiffness of the beam, which can be considered as the short-term stiffness under a static load, *B*_*nr*_ is the degradation stiffness of the beam after n times of the fatigue load, *B*_*Nr*_ is the degradation stiffness of the beam under the fatigue failure load, and *η*(*n*/*N*) is the stiffness degradation function of the beam under the fatigue load.

Formulas (1) or (2) is the formula for calculating the stiffness of the reinforced concrete beams under the fatigue load, for which initial stiffness *B*_*s*_ and failure stiffness *B*_*Nr*_ can be obtained by the experimental measurement of the deflection. Therefore, to obtain the stiffness of the beam under a fatigue load acting n times, the key is to determine stiffness degradation function *η*(*n*/*N*).

### 4.2 Determination of stiffness degradation function

According to the existing research results, the stiffness degradation function satisfies boundary conditions (a) and (b) and variation law described by (c) [10–11].

- The beam has no damage before fatigue loading, i.e., when *n*/*N* = 0, stiffness degradation function *η*(*n*/*N*) is 0 and the stiffness is the initial stiffness, *B*_*s*_.
- When the beam endures failure, the damage reaches the maximum, i.e., when *n*/*N* = 1, stiffness degradation function *η*(*n*/*N*) is 1 and stiffness is reduced to *B*_*Nr*_.
- The stiffness of the beam undergoes a relatively large decrease in the initial stage of the fatigue loading and last stage before the failure; however, in the middle for a long period time, the stiffness of the beam is basically in a linear development stage, i.e., in the entire process, the degradation of the stiffness exhibits a clear "S" shape.

It is easy to obtain a function that satisfies conditions (a) and (b), but variation law (c) is more difficult to satisfy. After a preliminary attempt, a function satisfying the above requirements was selected. Its formula is as follows:

$$\eta (\frac{n}{N})=1-\frac{1-{\left(\frac{n}{N}\right)}^{u}}{{\left(1-\frac{n}{N}\right)}^{v}}$$ (3)

Formula (3) is the function defined by Lian Wei [12] in the study of the stiffness of composite laminates, and it has achieved good results.

If we substitute formula (3) into formula (2), then formula (2) becomes

$$\frac{B_{nr}}{B_{s}}=1-\left(1-\frac{B_{Nr}}{B_{s}}\right)\left\{1-\frac{1-{\left(\frac{n}{N}\right)}^{u}}{{\left(1-\frac{n}{N}\right)}^{v}}\right\}$$ (4)

Formula (4) indicates that to study the stiffness degradation law of reinforced concrete beams under fatigue loading, the most important step is to determine the parameters of the stiffness degradation function.

### 4.3 Fatigue stiffness degradation law

In this study, the stiffness of the beam under different fatigue load cycles is obtained by the deflection measured by JL-2–JL-4 beams in the experiment. For this, the relationship between the deflection and stiffness of the reinforced concrete simple beam under a static load is used. Then, the parameters of the stiffness degradation function (formula (4)) are determined by a nonlinear fitting method. Finally, the stiffness degradation law of the experimental beam under fatigue loading is obtained.

#### 4.3.1 Stiffness calculated from deflection

According to material mechanics, the calculation formula of the deflection of a reinforced concrete simple beam under a short-term load is as follows:

$$f=\alpha \frac{ML^{2}}{B_{s}}$$ (5)

In formula (5), *α* is the deflection coefficient of the beam, which is related to the supporting condition and load form, *M* is the maximum bending moment of the mid-span cross-section of the beam, *L* is the calculated span of the beam, and *B*_*s*_ is the bending stiffness of the beam.

From formula (5), the expression of the bending stiffness of the beam is obtained as follows:

$$B_{s}=\alpha \frac{ML^{2}}{f}$$ (6)

Formula (6) is the stiffness expression of a reinforced concrete beam under a static load. Considering the experimental scheme of this study, according to the textbook on structural mechanics compiled by Long Yu-qiu [13], *α* can be taken as 23/216. The initial stiffness of the experimental beam is calculated by the deflection of the first fatigue loading. The stiffness of the reinforced concrete beams after n times of the fatigue load can be calculated by the following formula:

$$B_{nr}=\frac{23}{216}\times \frac{0.4Pl_{0}{}^{2}}{f_{n}}=0.283\frac{P}{f_{n}}$$ (7)

In formula (7), *P* is the upper limit of the fatigue load, *f*_*n*_ is the corresponding deflection at the time of loading to *P*, and *l*_0_ is the calculated span of the beam. The deflection presented in Table 4 measured by experiment is calculated according to formula (5). Finally, the fatigue stiffness values of JL-2–JL-4 beams, as listed in Table 5, can be obtained.

**Table 5:** Experimental stiffness results (*MN* ⋅ *m*^2^).

JL-2	cycle number	1	1000	1000	2000	50000	10000	20000	40000	60000	80000
	stiffness	9.874	8.687	8.102	7.568	6.907	6.416	5.976	5.605	5.239	4.996
	cycle numbers	100000	120000	140000	160000	168000					
	stiffness	4.788	4.483	4.256	3.818	3.697					
JL-3	cyclic number	1	1000	1000	2000	5000	10000	20000	40000	60000	80000
	stiffness	9.544	8.598	7.917	7.255	6.539	5.994	5.710	5.372	5.111	4.915
	cycle number	100000	120000	126000							
	stiffness	4.615	4.068	3.615							
JL-4	cycle number	1	1000	1000	2000	5000	10000	20000	40000	60000	62000
	stiffness	9.494	8.598	7.917	7.457	6.451	6.408	5.974	5.507	5.061	4.463

#### 4.3.2 Fatigue stiffness degradation analysis

**Fig 13:**
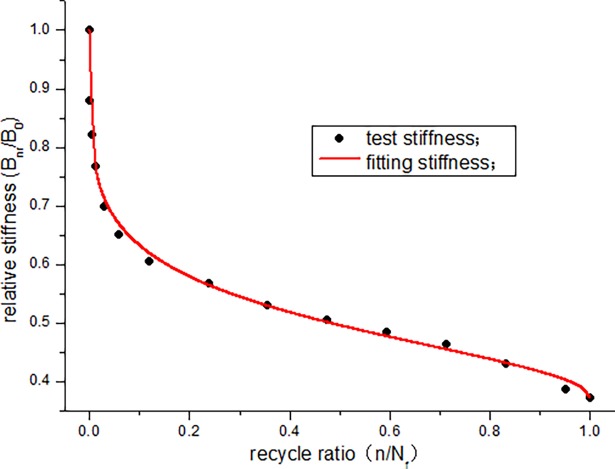
Stiffness curves of JL-2 beam.

**Fig 14:**
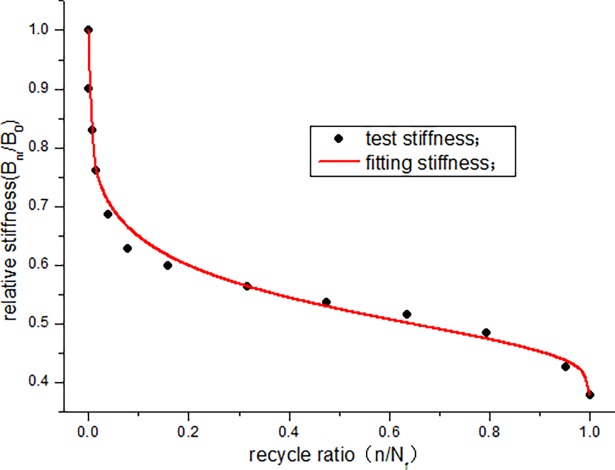
Stiffness curves of JL-3 beam.

**Fig 15:**
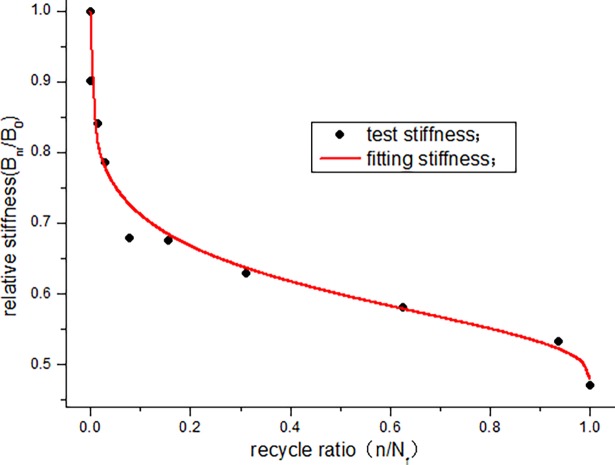
Stiffness curves of JL-4 beam.

According to the data in Table 5, the stiffness degradation of JL-2, JL-3, and JL-4 beams is 62.7%, 53.7%, and 47.7%, respectively, and the average degradation rate is approximately 54.7%. To study the degradation law of stiffness, the stiffness degradation function (formula (4)) is fitted with the data obtained from this experiment. To facilitate the calculation, first, a non-dimensional treatment for the stiffness data in Table 5 is conducted in the analysis. The curves obtained are shown in Figs 13–15. The parameters of the stiffness degradation function obtained from the fitting are listed in Table 6.

**Table 6:** Parameter values.

JL-2	u	v	fitting accuracy (R^2^)
	0.2171	0.4914	0.9840
JL-3	u	v	fitting accuracy (R^2^)
	0.2247	0.7044	0.9880
JL-4	u	v	fitting accuracy (R^2^)
	0.2362	0.6123	0.9753

From the fitting results of the stiffness degradation function parameters, we can see that the average precision of fitting is more than 0.98. Figs 13–15 show that the stiffness of the beam exhibits a relatively large decrease at the start of the fatigue loading. In particular, the stiffness of JL-2 and JL-3 beams experiences a large decrease when the number of fatigue cycles is less than 200000 times, and the stiffness of JL-4 beam decreases significantly when the number of fatigue cycles is less than 100000 times. At this instant, the fatigue cycle ratio of the three beams is less than 0.15. In the middle, for a long period, it is basically in a linear decline phase. Before the failure, the stiffness drastically decreases. Overall, the stiffness degradation trend is clearly "S" shaped.

From the fitting, it is found that parameter u can determine the initial degradation quantity and initial degradation rate of the stiffness degradation curve. Parameter v can reflect the degradation quantity and degradation rate of the near failure. The degradation rate of the intermediate stiffness is related to both u and v, and the change in the parameters can describe any type of stiffness degradation law. Therefore, the stiffness degradation function (formula (4)) can accurately express the stiffness degradation law.

## 5. Bearing capacity degradation law

### 5.1 Definition of fatigue bearing capacity

From the definition of the fatigue stiffness, the expression for the degradation of the bearing capacity under a fatigue load can be defined as

$$S_{nr}=S_{0}-\left(S_{0}-S_{Nr}\right)g\left(\frac{n}{N}\right)$$ (8)

$$\frac{S_{nr}}{S_{0}}=1-\left(1-\frac{S_{Nr}}{S_{0}}\right)g\left(\frac{n}{N}\right)$$ (9)

In formulas (8) and (9), *S*_0_ is the initial bearing capacity of the beam, *S*_*nr*_ is the degradation bearing capacity of the beam after n times the fatigue load, *S*_*Nr*_ is the degradation bearing capacity of the beam under the fatigue failure load, and *g*(*n*/*N*) is the bearing capacity degradation function of the beam under fatigue loading.

It is found that the bearing capacity degradation function satisfies the following boundary conditions:

- The beam has no damage before fatigue loading, i.e., when *n*/*N* = 0, the bearing capacity degradation function *g*(*n*/*N*) is zero and the bearing capacity is the initial bearing capacity, *S*_0_, which can be obtained from a static load test, numerical simulation, or theoretical calculation.
- When the beam endures a failure, the damage reaches the maximum, i.e., when *n*/*N* = 1, the bearing capacity degradation function *g*(*n*/*N*) is 1 and the bearing capacity is reduced to *S*_*Nr*_, which can be considered as the maximum loading value during the fatigue test, *M*_max_.

### 5.2 Fatigue damage based on stiffness degradation

The stiffness degradation of reinforced concrete beams is the external expression for the structural damage, which reflects the degree of the structural damage to some extent. Moreover, it is easy to test the stiffness during fatigue. Therefore, the stiffness can be used as an index to decide the damage degree of the structure. Based on the establishment of the stiffness formula of the reinforced concrete beam under fatigue loading, the relationship between the stiffness and structural damage is established as follows:

$$D_{B}=\frac{B_{s}-B_{nr}}{B_{s}-B_{Nr}}$$ (10)

In formula (10), *D*_B_ is the damage variable defined by the stiffness and *D*_B_ ∈ [0,1].

### 5.3 Fatigue damage based on bearing capacity degradation

Similar to stiffness degradation, the degradation of the bearing capacity is another form of structural damage. Based on the results of Fong [14] in the study of the fatigue damage of composite materials, three hypotheses are proposed in this paper:

- The material is damaged during fatigue loading, and both the stiffness and bearing capacity of the material decrease.
- With the increase in the number of fatigue loads, the fatigue damage is accumulated, which is an irreversible process, and the stiffness and bearing capacity of the material decrease monotonically with the increase in the damage.
- At the same time of fatigue, the damage is in the same state; however, the stiffness and bearing capacity have different forms, which are determined by the damage state of the material. Therefore, there is a certain relationship between the stiffness damage and bearing capacity damage occurring at the same time.

Based on these three assumptions and combined with the fatigue damage defined by the stiffness degradation, it is possible to define the damage caused by the bearing capacity degradation under fatigue loading. This is defined as follows:

$$D_{S}=\frac{S_{0}-S_{nr}}{S_{0}-S_{Nr}}$$ (11)

In formula (11), *D*_S_ is the damage variable defined by the bearing capacity and *D*_S_ ∈ [0,1].

### 5.4 Relationship between stiffness and bearing capacity

It is found that the damages based on stiffness and bearing capacity have the following characteristics:

- The range of both *D*_B_ and *D*_S_ is [0,1].
- The value of both damage *D*_B_ and *D*_S_ is 0 at the start of the fatigue, and is 1 at the time of fatigue failure.
- Both *D*_B_ and *D*_S_ are monotone increasing functions in interval [0,1].

Through the analysis of the above characteristics and combination of assumption (c) in section 5.3, the relationship between the two damages can be defined as

$$D_{S}={\left(D_{B}\right)}^{p}$$ (12)

If we substitute formula (10) of *D*_B_ and formula (11) of *D*_S_ into formula (12), then formula (15) becomes

$$\frac{S_{0}-S_{nr}}{S_{0}-S_{Nr}}={\left(\frac{B_{s}-B_{nr}}{B_{s}-B_{Nr}}\right)}^{p}$$ (13)

After processing formula (13), we obtain

$$S_{nr}=S_{0}-\left(S_{0}-S_{Nr}\right){\left(\frac{B_{0}-B_{nr}}{B_{0}-B_{Nr}}\right)}^{p}$$ (14)

First, a simple transformation of formula (4) is shown as follows:

$$\frac{B_{0}-B_{nr}}{B_{0}-B_{Nr}}=1-\frac{1-{\left(\frac{n}{N}\right)}^{u}}{{\left(1-\frac{n}{N}\right)}^{v}}$$ (15)

Then, substituting formula (15) into formula (14) yields

$$S_{nr}=S_{0}-\left(S_{0}-S_{Nr}\right){\left(1-\frac{1-{\left(\frac{n}{N}\right)}^{u}}{{\left(1-\frac{n}{N}\right)}^{v}}\right)}^{p}$$ (16)

or

$$\frac{S_{nr}}{S_{0}}=1-\left(1-\frac{S_{Nr}}{S_{0}}\right){\left(1-\frac{1-{\left(\frac{n}{N}\right)}^{u}}{{\left(1-\frac{n}{N}\right)}^{v}}\right)}^{p}$$ (17)

Formulas (16) or (17) is the expression of the relationship between the stiffness degradation and bearing capacity degradation. From the experimental data of the fatigue stiffness (obtained by testing the deflection), parameters *u*, *v* in the stiffness degradation expression are easily determined. Parameters *u*, *v* in the expression of the bearing capacity degradation can be the same as those in the expression of stiffness degradation, and the effect of life ratio *n*/*N* on the stiffness and bearing capacity can be reflected by critical parameter *p*, which can be determined at least through an experimental data point of the residual bearing capacity. The workload and cost also can be reduced.

### 5.5 Bearing capacity degradation law

#### 5.5.1 Solving the correlation parameters

Because JL-4 and JL-5 beams are in the same loading state, they can be used to solve the correlation parameters. In this experiment, *S*_0_ is 220 kN, *S*_*Nr*_ is 176 kN, *S*_*nr*_ is 180 kN, and *u*,*ν* are 0.2362 and 0.6123, respectively. Substituting the above parameter values into formula (19), we obtain

$$\frac{180}{220}=1-\left(1-\frac{176}{220}\right){\left(1-\frac{1-{\left(\frac{50}{64}\right)}^{0.2362}}{{\left(1-\frac{50}{64}\right)}^{0.6123}}\right)}^{p}$$ (18)

Finally, calculated correlation parameter *p* is 0.6556. It should be noted that in this experiment, the fatigue lower limit of JL-2–JL-4 beams is zero and fatigue upper limit is 0.6*P*_*u*_, 0.7*P*_*u*_, and 0.8*P*_*u*_, respectively. Parameter p is obtained by the combination of JL-4 and JL-5 beams. Because of the limitation of the subjective and objective conditions such as the capital, a corresponding failure test of JL-2 and JL-3 beams was not performed to determine their correlation parameters. Therefore, in this study, parameter p acquired from JL-4 and JL-5 beams is still used in the analysis of the bearing capacity degradation of JL-2 and JL-3 beams.

#### 5.5.2 Bearing capacity degradation law

The initial capacity of JL-2–JL-4 beams can be determined using the initial load and position of the distribution beam in the experiment. It is calculated as 220/2×1.1 = 121*kN* ⋅ *m*. Similarly, the bearing capacity of each beam at the failure time is 72.6*kN* ⋅ *m*, 84.7*kN* ⋅ *m*, and 96.8*kN* ⋅ *m*, respectively.

**Fig 16:**
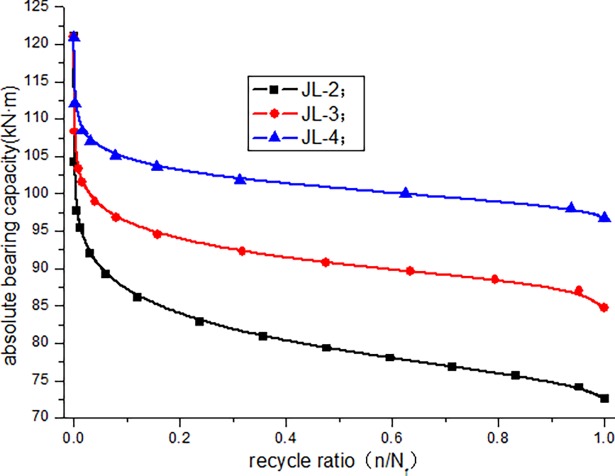
Bearing capacity degradation curves of JL-2–JL-4.

**Fig 17:**
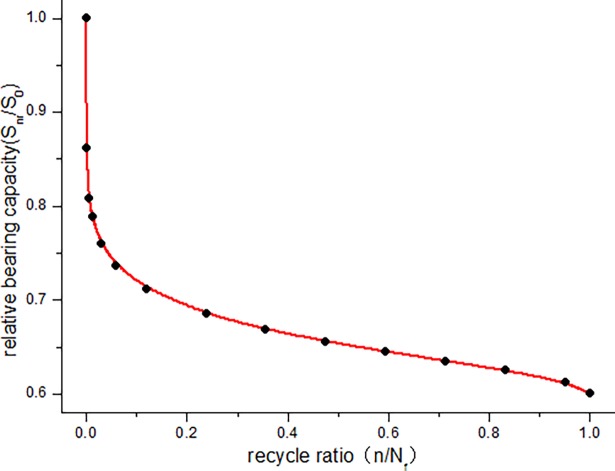
Relative bearing capacity degradation curve of JL-2 beam.

**Fig 18:**
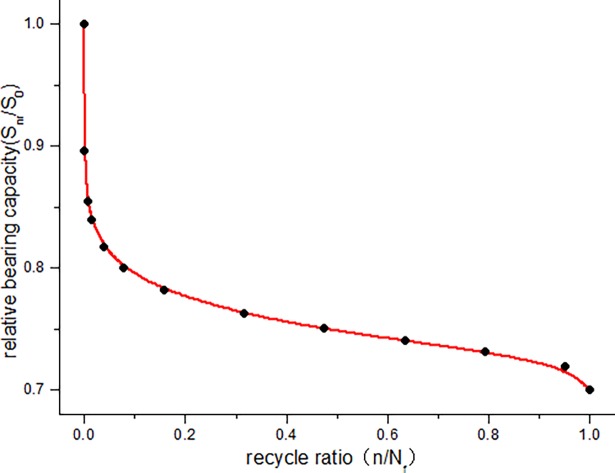
Relative bearing capacity degradation curve of JL-3 beam.

**Fig 19:**
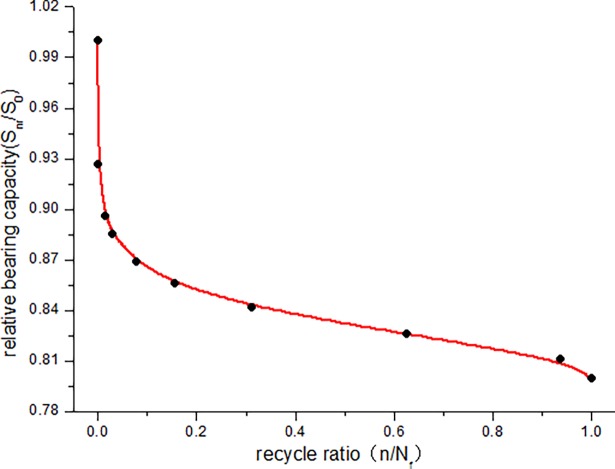
Relative bearing capacity degradation curve of JL-4 beam.

The calculated bearing capacity degradation law of JL-2–JL-4 beams under fatigue load is shown in Fig 16. The calculated relative bearing capacity degradation law of JL-2–JL-4 beams under fatigue load is shown in Figs 17–19.

Fig 16 clearly depicts the absolute capacity degradation. When the cycle number is zero, the bearing capacity of the beam is equal to the initial capacity (static load bearing capacity), which is 121*kN* ⋅ *m* in this study. When the number of cycles reaches the fatigue life, the residual bearing capacity of the beam is reduced to the maximum experimental load, i.e., JL-2 beam is 72.6*kN* ⋅ *m*, JL-3 beam is 84.7*kN* ⋅ *m*, JL-4 beam is 96.8*kN* ⋅ *m* in this paper. This is consistent with the bearing degradation boundary condition. It can be seen from the absolute bearing capacity degradation in Fig 16 and relative bearing capacity degradation in Figs 17–19 that, the bearing capacity of the beam undergoes a relatively large decrease at the start of the fatigue loading. In the middle, for a long period, it exhibits a linear decline phase, and before the failure, the bearing capacity significantly decreases. Overall, the bearing capacity degradation trend has a clear "S" shape, which is consistent with the stiffness degradation.

## 6. Conclusion

1. From fatigue test, it was found that the stiffness degradation of the reinforced concrete beam exhibits a very clear monotonic decreasing "S" curve. At the initial stage of the fatigue loading, the stiffness degradation of the beam is very clear After 0.15 *N*_f_, the stiffness degradation rate of the beam is very small, which is close to a constant. After 0.9 *N*_f_, the stiffness degradation rate increases gradually, and the test beam enters the stage of fatigue fracture and is shortly destroyed.
2. By constructing a function and fitting the experimental data, a formula used for calculating the stiffness degradation of reinforced concrete beams is obtained. The result of the formula is in good agreement with the experimental result, which can be used to quantitatively describe the stiffness degradation.
3. The stiffness degradation and bearing capacity degradation of the reinforced concrete beams are external manifestations of the structural damage. In this study, based on the assumption that the residual stiffness and residual bearing capacity depend on the same damage state, the relationship between the residual stiffness and residual bearing capacity is determined, and then the bearing capacity degradation model of the reinforced concrete beams based on the fatigue stiffness degradation is established. Using the established model and under the premise of the known residual stiffness degradation law, the degradation law of the bearing capacity is determined by using at least one residual bearing capacity test data, for which the parameters of the stiffness degradation are used as material constants.
4. The bearing capacity of the beam undergoes a relatively large decrease at the start of the fatigue loading. In the middle, for a long period, it is basically in a linear decline phase. Before the failure, the bearing capacity decreases significantly. Overall, the bearing capacity degradation process has an obvious "S" shape, which is consistent with the stiffness degradation process.
5. The stiffness degradation expression and the expression of bearing capacity degradation based on the stiffness degradation are used to determine the degree of deterioration of reinforced concrete beams. In particular, the expression of the bearing capacity degradation can avoid numerous destructive tests and save cost. The results obtained in this study can be used to calculate the deformation and residual bearing capacity of reinforced concrete beams and crane beams subjected to dynamic loads. It can also provide reference for engineering structure maintenance decision.
